# Farm level survey of spore‐forming bacteria on four dairy farms in the Waikato region of New Zealand

**DOI:** 10.1002/mbo3.457

**Published:** 2017-03-03

**Authors:** Tanushree B. Gupta, Gale Brightwell

**Affiliations:** ^1^ Food Assurance and Meat Quality Hopkirk Research InstituteAgResearch Limited, Massey University Campus Palmerston North New Zealand

**Keywords:** *bacillus*, *clostridium*, dairy farm, spores

## Abstract

The aim of our study was to determine the occurrence and diversity of economically important spore‐forming bacteria in New Zealand dairy farm systems. Farm dairy effluent (FDE) collected from Waikato dairy farms were tested for the presence of spore‐forming bacteria, using a new culture‐based methodology followed by genomic analysis. An enrichment step in which samples were inoculated in cooked meat glucose starch broth under anaerobic conditions, aided in the differential isolation of *Bacillus* and *Clostridium* species. Furthermore, the use of molecular methods such as ERIC genotyping, 16S rRNA gene sequence analysis identified different spore‐forming bacteria present in FDE. *C. sporogenes* signature PCR gave further information on the phylogenetic relationship of the different *Clostridium* spp. isolated in this study. In total 19 *Bacillus* spp., 5 *Paenibacillus* spp. and 17 *Clostridium* spp. were isolated from farm dairy effluent. Sequence types similar to economically important food spoilage bacteria viz: *C. butyricum*,* C. sporogenes* and members of the *Paenibacillus* Genus were isolated from all four farms, whereas, sequence types similar to potential toxigenic, *B. cereus, C. perfringens, C. butyricum*,* and C. botulinum* were found on at least three of the farms. Sampling of farm dairy effluent provides a good indicator of farm level prevalence of bacterial load as it is used to irrigate dairy pasture in New Zealand. This study highlights the presence of various spore‐forming bacteria in dairy waste water and indicates the implementation of good hygienic farm practices and dairy waste effluent management.

## 1 Introduction

Spores from bacteria such as *Bacillus* and *Clostridium* spp. may contaminate foods and are often responsible for food quality and safety issues. These spores are heat tolerant and pasteurization has little or no effect on their control. Currently good hygiene management and (thermal) processing largely control spore‐forming bacteria during food processing, ensuring quality, and safety of final products. Concerns have been raised regarding the emergence and description of highly heat‐resistant endospores (Foschino, Galli, & Ottogali,^1990^; Klijn et al.,^1997^) that can survive and grow after commercial sterilization and ultra‐high temperature (UHT) processing of milk. Also, mild heat treatments, such as thermization, may intensify problems by activating spore germination in that temperature (Griffiths, Phillips, West, & Muir,^1988^; Hanson, Wendorff, & Houck, 2005). Clonally related highly heat‐resistant spores of *Bacillus sporothermodurans* have been isolated from the farm environment as well as from heat‐treated milk highlighting the importance of the environment on spore resistance, survival and germination properties (Gerhardt & Marquis, 1989; Guillaume‐Gentil et al., 2002; Scheldeman, Pil, Herman, De Vos, & Heyndrickx, 2005). As new products and technologies have been developed, concerns have also been raised around the impact of spore contamination on quality and safety in minimally processed and shelf stable dairy foods (Guinebretiere, Girardin, Dargaignaratz, Carlin, & Nguyen‐The, 2003; Peck, 2006; Ranieri, Huck, Sonnen, Barbano, & Boor, 2009).

*Bacillus* and *Paenibacillus* spores can withstand high temperature short time (HTST) pasteurization, a common method used for raw milk processing (Fromm & Boor, 2004; Ranieri et al., 2009). *Bacillus* is often the predominant genus present postpasteurization in milk stored at 6°C (Huck, Woodcock, Ralyea, & Boor, 2007). Conversely *Paenibacillus* spp. usually dominate later on during chilled storage (Fromm & Boor, 2004; Ranieri et al., 2009) and have been found to comprise over 95% of the bacterial microflora present in milk after prolonged refrigeration (Ranieri et al., 2009, 2012). *Geobacillus stearothermophilus* is another species of significance to the dairy industry (Burgess, Lindsay, & Flint, 2010). This can cause long‐term persistent contamination of various dairy processing facilities, due to their strong ability to form biofilms on stainless steel surfaces of processing equipment in milk plants (Flint, Bremer, & Brooks, 1997). The metabolic activities of these spore formers can lead to curdling and off‐odors, or ‐flavors (Ageitos, Vallejo, Sestelo, Poza, & Villa, 2007; Dutt, Gupta, Saran, Misra, & Saxena, 2009).

Some of the Clostridia strains are associated with late blowing cheese, *C. sporogenes*,* C. beijerinckii*,* C. butyricum*, and *C. tyrobutyricum* being the major agents (Cocolin, Innocente, Biasutti, & Comi, 2004; Cremonesi, Vanoni, Silvetti, Morandi, & Brasca, 2012). *C. sporogenes* and *C. butyricum* can cause gassy defects of processed cheese. Moreover, if extensive proteolysis occurs during cheese ripening, the release of amino acids and increase in pH will favor the growth of many *Clostridium* species, especially *C. tyrobutyricum* (Klijn, Nieuwendorf, Hoolwerf, van derWaals, & Weerkamp, 1995). Silage has been implicated as the principal source of spores of ruminant feed (Vissers, Driehuis, Te Giffel, De Jong, & Lankveld, 2007a,b) and according to a study by Klijn et al. in 1995, *C. tyrobutyricum* was found to be one of the commonly isolated *Clostridium* spp. in silage samples. Other *Clostridium* species commonly found in silage include *C. beijerinckii* and *C. acetobutylicum*. Importantly, the reduction or elimination of these bacteria can result in an extended shelf life for pasteurized milk providing an overall higher quality product.

Human food poisoning caused by spore‐forming bacteria are often associated with heat‐treated foods subjected to mishandling and temperature abuse during storage, which results in spore germination, bacterial multiplication, and food consumption with hazardous levels of cells or toxins. For *Bacillus* spp., *B. cereus* is considered the most important human pathogen because of the ability of some strains to produce toxins (Ghelardi et al., 2002; Turnbull et al., 2002). Common *Clostridium* pathogenic species include *C. perfringens*,* C. difficile*,* C. botulinum*, with *C. perfringens* being the most commonly associated with raw milk products (McAuley, McMillan, Moore, Fegan, & Fox, 2014). The majority of the *Clostridium* spp. is of relevance to the dairy industry possess the metabolic ability to reduce sulphite to sulphide under anaerobic conditions and are termed as sulphite reducing Clostridia (SRCs) (Prevot, 1948; Weenk, Van den Brink, Struijk, & Mossel, 1995). SRCs are often used by the dairy industry as a hygienic indicator (Dodds, 1993; Aureli & Franciosa, 2002; New Zealand Ministry of Primary Industries (MPI), 2014) but the reduction of sulphite to sulphide is not a discriminatory factor between species.

Very little is known about the impact of farm management practices on the relative abundance and diversity of spore‐forming bacteria in dairy farm systems. The dairy industry relies on pasteurization to control the number of pathogenic and spoilage microorganisms. As pasteurization is relatively ineffective against spores (Griffiths & Phillips, 1990) it is important to understand how on farm practices may increase or reduce risk for raw bulk milk contamination. Silage type and quality have been implicated as the major primary source of spore‐forming bacteria in bulk tanker milk (Garde, Gaya, Arias, & Nuñez, 2012; Julien, Dion, Lafreniere, Antoun, & Drouin, 2008; Vissers, Te Giffel, Driehuis, De Jong, & Lankveld, 2007; Vissers et al., 2007a,b). Other factors that may influence silage quality include the starting material, pH, dry matter content, fermentation conditions and microbial content (Rammer, 1996; Vissers et al., 2007a,b); Vissers, Te Giffel, et al 2007. Current thinking is that raw milk becomes contaminated through consumption of lower grade silage by herd, followed by the survival of spores in the bovine gastrointestinal tract resulting in contaminated faeces. Subsequent fecal contamination of teats and udder surfaces then result in contamination of raw milk particularly if good hygienic practice is not followed (Aureli & Franciosa, 2002). Also, the teats and udders of pasture fed cows can become contaminated through spore‐forming bacterial contaminants of soil, particularly *Clostridium* species (Christiansson, Bertilsson, & Svensson, 1999; Slaghuis, Te Giffel, Beumer, & André, 1997).

Whether the contamination of bulk milk with spore‐forming bacteria can be eliminated is unknown. Annually, New Zealand dairy companies process around a billion litres of milk, most of which will go overseas as whole milk powder to be used globally as ingredients or for the production of infant formula. If studies to investigate the diversity of spore‐forming bacteria and their prevalence on farm are to generate mitigation strategies to control their entry to the food chain, simple tools for their rapid detection and differentiation are required. This study aimed at investigating diversity of spore‐forming bacteria in farm dairy effluent (FDE) as cultures from dairy effluent provide an indication of microbial strains that may be cycling on the dairy farm. We developed a new culture based methodology to detect and separate *Clostridium* and *Bacillus* species anaerobically. To date, we believe that no study has been undertaken to investigate the occurrence of spore‐forming bacteria at a farm level in New Zealand.

## 2 Materials and Methods

### 2.1 Study sites

A cross‐sectional pilot study was carried out on four Waikato dairy farms. All the 4 study farms had some component of pasture grazing; three were pasture‐only and in one, the cows had access to HerdHomes^®^‐like facility (http://herdhomes.co.nz). Analysis was carried out on a “whole herd” basis by collecting samples of FDE from the exit point of the milking parlor at final wash down.

### 2.2 Sample processing

Farm dairy effluents were collected in July and September 2014 (late winter early spring) and in January 2015 (summer). A one litre grab sample of FDE was collected in a sterile Schott Duran glass bottle from the shed wash collection sump immediately after morning milking. Samples were transported to the laboratory in an insulated box and kept frozen until processed. For ease of recording and to retain confidentiality of individuals, farms were allocated a number.

Twenty ml of farm dairy effluent was dissolved in 250 ml of prewarmed Butterfield's diluents (Fort Richard, New Zealand) and centrifuged at 3466*g* for 1 hr. The supernatant was removed and pellet suspended in 5 ml of prewarmed Butterfield's diluent. To isolate spores from vegetative cells, the respective suspensions were heated at 80°C for 15 min in a water bath.

### 2.3 Bacterial isolates

*Clostridium sporogenes* NCTC 532 spores were used as a control and to develop the new methodology.

### 2.4 New culture‐based methodology for isolating mesophilic spore‐forming bacteria from environmental samples

#### 2.4.1 Aerobic spore formers

A 1 ml aliquot from each of the heated sample suspensions described previously was serially diluted in Butterfield's diluent and plated directly in duplicate on Sheep Blood Agar (SBA) (Fort Richard, New Zealand). Plates were incubated under aerobic conditions at 35°C for 24 hr and then colonies enumerated. This preparation was termed, heated and direct (HD).

#### 2.4.2 Anaerobic spore formers

A 1 ml aliquot of each of the heated sample suspensions was added to 9 ml of prereduced cooked meat glucose broth (Fort Richard) supplemented with casein (0.03%), L‐cysteine (0.0005%), Haemin (0.1%), Vitamin K1 (1%) and yeast extract (0.0005%) for sample enrichment and incubated under anaerobic conditions at 35°C for 3 day. This treatment was termed, heated and enriched (HE). Enriched cultures were removed and transferred to fresh sterile centrifugation tubes. The samples were centrifuged at 6000*g* for 15 min and the pellets were re‐suspended in 2 ml of Butterfield's diluent. A 1 ml preparation from each of the enriched suspensions was serially diluted in Butterfield's diluent and plated in duplicate on Shahidi‐ Ferguson Perfringens agar (Shahidi & Ferguson, 1991) with 50% egg yolk, polymyxin B (3 mg l^−1^) and kanamycin (12 mg l^−1^) (EYA). Plates were incubated under anaerobic conditions at 35°C for 24–48 hr. Enumeration of anaerobic spore formers was not carried out as the samples were enriched in growth medium for 3 days.

All the colonies from both HD, and HE plates (black as well as white colonies on EYA plates) were further sub cultured on Sheep blood agar for obtaining pure cultures. Each of the cultures was further inoculated in thioglycollate broth (Fort Richard) for genomic analysis.

### 2.5 DNA extraction

A boiled lysate of each of the cultures was prepared by boiling the culture at 100°C for 10 min and collecting the supernatant after centrifugation at 9838*g* for 5 min. The supernatant was used as a source of template DNA for Enterobacterial Repetitive Intergenic Consensus (ERIC) PCR. Genomic DNA was isolated, using the Roche High Pure PCR template preparation kit (Roche diagnostics, Manheim, Germany), according to the manufacturer's instructions and used for amplification of the 16S rRNA gene from spore‐forming bacteria.

### 2.6 ERIC PCR genotypic fingerprinting

ERIC PCR was carried out using ERIC 2 primer (Versalovic, Koueth, & Lupski, 1991) and the protocol of Weijtens, Reinders, Urlings, and Van der Plas (1999). The PCR reaction mixtures (50 μl) had 1 ×  Amplitaq Gold (Applied Biosystems, Melbourne, Australia), 1 μmol/L of ERIC 2 primer with sequence 5′‐ AAG TAA GTG ACT GGG GTG AGC‐3′ (Invitrogen, Thermo Fisher Scientific Northshore, City, NZ) and 5 μl of extracted DNA. The PCR protocol was an initial cycle of 5 min at 94°C, 40 cycles of consecutively 1 min at 94°C, 1 min at 25°C and 4 min at 72°C and finally, a cycle of 10 min at 72°C. PCR was carried out in a PTC‐100^™^ Programmable Thermal Cycler (MJ Research Inc, Thermo Fisher Scientific). PCR products of 1500 bp were separated on a 1.5% agarose gel (Fisher Scientific, Loughborough, UK), stained with ethidium bromide (10 mg l‐1 Bio‐Rad Laboraotories, North Harbour, NZ), at 150 V for 4 hr and visualized under UV transillumination using Gel DocTM XR+ (Bio‐Rad Laboratories) and images of band patterns (fingerprints) were captured, using Image LabTM software version 3.0 (Bio‐Rad Laboratories). The fingerprints were compared, using Quanti‐One software (v. 4.5.2 Bio‐Rad), with the background subtraction level set at a rolling disk size 2 and a minimum spacing between bands (tolerance) set at 1% of the lane height. All bands were included in the fingerprint comparison. Each unique ERIC profile for each sequence type was allocated a letter of the alphabet and genomic DNA from representative ERIC profiles was chosen for 16S rRNA gene analysis.

### 2.7 16S rRNA gene amplification and Sequencing

Amplification of the 16S rRNA gene was carried out using forward primer sequence pA 5′‐AGAGTTTGATCCTGGCTCAG‐3′ (Invitrogen) and reverse primer sequence pH* 5′‐ AAGGAGGTGATCCAGCCGCA‐3′ (Invitrogen) as described by Boddinghaus, Wolters, Heikens, and Bottger (1990). Pure DNA isolated from the Roche High Pure PCR template preparation kit was used for the reaction. Each 50 μl PCR mixture contained 0.2 mmol/L of each dNTPs (Invitrogen), 1 μmol/L of forward and reverse primers, 1X reaction buffer (Invitrogen) with 2 mmol/L of MgCl2 (Invitrogen), 1.25 U of Taq polymerase (Invitrogen) and 5 μl of DNA. All the reagents were procured from Life Technologies and nuclease free water was used in all the reactions. PCR was carried out in a PTC‐100^™^ Programmable Thermal Cycler (MJ Research Inc), using the following conditions: 93°C for 3 min; 92°C for 1 min, 55°C for 1 min and 72°C for 2 min for 30 cycles followed by a final extension at 72°C for 3 min. PCR products were visualized on a 0.8% ultrapure agarose (GibcoBRL) gel stained with ethidium bromide (Bio‐Rad, 10 mg l‐1). The PCR products were purified, using Qiagen DNA extraction kit as per manufacturer's instructions (Qiagen, Bio‐strategy Ltd, New Zealand) and the products sequenced, using an ABI3730 DNA Analyzer (Massey Genome Service, Palmerston North, New Zealand) and the primers described above. 16S rRNA gene consensus sequences were used to investigate the phylogenetic relationship of the spore‐forming bacteria obtained from the samples, using the software Geneious version 8.1 by Biomatters. (http://www.geneious.com, Kearse et al., 2012).

### 2.8 Phylogenetic analysis

Phylogenetic analysis was carried out, using the software Geneious version 8.1 by Biomatters. A Polar formatted, unrooted phylogenetic tree was created from the converted 16s rRNA gene sequence alignment of all the representative isolates identified during this study and botulinum Group I, II and III to investigate genetic distance between the sequences. 16S rRNA gene sequences of botulinum Groups I, II and III were obtained from Genbank (http://www.ncbi.nlm.nih.gov/genbank/) and PATRIC (Pathosystems Resource Integration Centre, https://www.patricbrc.org). Alignment of all 16S rRNA gene sequences was prepared, using Muscle alignment tool with UPGMA clustering. The most likely tree found using a General Time Reversible model with optimized nucleotide equilibrium frequencies, optimized invariable sites, optimized across site variation, and NNI tree‐searching operations was bootstrapped with 100 replicates. Branches with less than 50% support were collapsed, using TreeCollapserCL version 4 (Hodcroft, 2013)

### 2.9 *C. sporogenes* genetic signature PCR

PCR primers to *hsdMSR* (Forward 5′‐TGAATCGGAAACCGATGGAC‐3′; Reverse 5′‐ TGCCACTTGGCTTCATTTCT‐3′)*, thiHG* (Forward 5′‐ RCGCTTGTGTCCAKCTAAAT‐3′; Reverse 5′‐ GCCGGGTATGGAGYTAATTG‐3′)*, lipAS* (Forward 5′‐ TGTGACGAAGCTAATTGTCCT‐3′; Reverse 5′‐ CTTTTTCCCCAAGACCTACCA‐3′) and *dapL* (Forward 5′‐ ACGCCTTCTGTAGCATCAAA‐3′; Reverse 5′‐ GCCTGGACTTTAGGTACTGC‐3′) gene sequences were used in this study (Weigand et al., 2015). Each 25 μl PCR mixture contained 0.2 mmol/L of each dNTPs (Invitrogen), 1 μmol/L of forward and reverse primers, 1X reaction buffer (Invitrogen) with 1 mmol/L of MgCl_2_ (Invitrogen), 1.25 U of Taq polymerase (Invitrogen) and 2 μl of DNA. All reagents were procured from Life Technologies and nuclease free water was used in all the reactions. PCR amplification was carried out in a T100^™^ Bio‐Rad Thermal Cycler, using the following conditions: 95°C for 3 min; 95°C for 30 s, 53°C for 30 s and 72°C for 90 s for 35 cycles followed by a final extension at 72°C for 10 min. PCR products were visualized on a 2% ultrapure agarose (GibcoBRL) gel stained with ethidium bromide (Bio‐Rad, 10 mg l^−1^).

## 3 Results

### 3.1 Culture experiments

Mesophilic spore‐forming bacteria were isolated from all FDE samples collected from the four farms during all sampling events. The new methodology and conditions described in this study successfully differentiated *Bacillus* and *Clostridium* spp. on culture medium in anaerobic conditions. The use of an enrichment step (using prereduced cooked meat glucose broth vials supplemented with casein (0.03%), L‐cysteine (0.0005%), Haemin (0.1%), Vitamin K1 (1%) and yeast extract (0.0005%) prior to plating on Shahidi‐ Ferguson Perfringens Agar supplemented with 50% egg yolk and polymyxin B (3 mg l^−1^) and kanamycin (12 mg l^−1^) under anaerobic conditions aided the isolation of *Clostridium* spp. and inhibited the growth of facultative anaerobic *Bacillus* spp.

Enumeration and isolation of mesophilic aerobic spore‐forming bacteria were carried out by direct plating of heat treated sample suspensions serially diluted in Butterfield's diluent and plated on Sheep Blood Agar and incubated under aerobic conditions at 35°C for 24 hr. Farm 1, did not show any difference in number of colony‐forming units ml^−1^ in winter (20 × 10^3^ CFU ml^−^1, July 14) and summer (23 × 10^3 ^CFU ml^−^1, Jan 15). Farm 2 had 25 × 10^5^ in winter (July 14) and 38 × 10^4 ^CFU ml^−1^ in summer (Jan 15), Farm 3 had 21 × 10^5^ in winter (July 14) and 28 × 10^3 ^CFU ml^−1^ in summer (Jan 15) whereas, Farm 4 did not show huge difference between two seasons; 8 × 10^3^ July 14 and 28 × 10^3^ Jan 15 CFU ml^−1^, respectively (Table 3).

### 3.2 ERIC PCR genotypic fingerprinting and 16S rRNA sequencing

ERIC PCR was able to differentiate between isolates cultured from all FDE samples which aided in selecting unique representatives for 16S rRNA gene sequencing (Tables 1 and 2). The maximum identity for the 16S rRNA gene sequences obtained in this study in comparison to type strains ranged from 92 to 100% (Tables 1 and 2) therefore, species were identified on the basis of closest‐related taxonomically described species.

**Table 1:** Diversity of aerobic spore‐forming bacteria isolated from four Waikato farms over two seasons

Farm, Seasons	Eric Type^b^	Number of isolates	Closest‐related taxonomically described species	Maximum Identity (%)^a^	GenBank Accession Number
Farm 1, Winter	A	7	*B. licheniformis* (T); ATCC 14580	98.4	CP000002
	B	3	*B. circulans* ATCC 4513]	99.6	FJ560956
	C	2	*B. pumilus* ATCC 7061]	99.6	AY876289
	D	1	*B. thuringiensis* IAM 12077	99.1	D16281
	E	2	*Paenibacillus lactis* strain MB 1871	99.7	NR_025739
	F	1	*Virgibacillus proomii* LMG 12370	99.7	AJ012667
	G	1	*B. clausii* (T)DSM 8716	99.3	X76440
	H	1	*B. siralis* (T) 171544	99.6	AF071856
	I	1	*B. thermoamylovorans* TN20	98.1	JQ415992
	J	1	*B. mycoides* ATCC6462	99.2	AB021192
Farm 1, Summer	A	1	*B. licheniformis* (T); ATCC 14580	97.8	CP000002
	B	3	*B. pumilus* (T); ATCC 7061	99.7	AY876289
	C	1	*B. megaterium* (T); IAM 13418	97.4	D16273
	D	1	*B. circulans* (T); ATCC 4513	99.4	AY724690
	E	1	*B. cereus* (T); ATCC 14579	97.4	AE016877
	F	2	*Paenibacillus cookii* strain JGR8	99.7	KF873508
	G	5	*Paenibacillus cookii* (T); LMG 18419	99.4	AJ250317
	H	1	*Brevibacillus agri* (T); DSM 6348T	95.1	AB112716
	I	7	*B. altitudinis* (T); type strain:41KF2b	99.6	AJ831842
	J	1	*Solibacillus silvestris* StLB046	99.5	AP012157
Farm 2, Winter	A	21	*B. licheniformi*s (T); ATCC 14580; DSM 13	98.8	CP000002
	B	2	*B. circulans* ATCC 4513	99	AY724690
	C	2	*B. clausii* (T); DSM 8716	98.7	X76440
Farm 2, Summer	A	18	*B. licheniformis* (T); ATCC 14580	99.6	CP000002
	B	4	*B. licheniformis* (T); ATCC 14580	100	CP000002
	C	1	*B. licheniformis* (T); ATCC 14580	99.6	CP000002
	D	1	*B. licheniformis* (T); ATCC 14580	97.6	CP000002
	E	1	*B. licheniformis* (T); ATCC 14580	99.6	CP000002
	F	1	*B. circulan*s strain ATCC 4513	99.0	NR_104566
	G	2	*B. subtilis* (T); DSM10	100	AJ276351
	H	2	*B. cereu*s (T); ATCC 14579	97.6	AE016877
	I	1	*B. thuringiensis* (T); IAM 12077	99.6	D16281
	J	3	*B. amyloliquefacien*s (T); DSM7	99.1	FN597644
	K	1	*Paenibacillus lactis* (T); MB 1871	97.5	AY257868
	L	1	*Sporosarcina koreensis* (T); F73	95.4	DQ073393
	M	1	*B. shackletonii* (T); LMG 18435	92	AJ250318
	N	1	*B. idriensis* (T); SMC 4352‐2	97.4	AY904033
Farm 3, Winter	A	2	*B. licheniformi*s (T); ATCC 14580; DSM 13	98.3	CP000002
	B	2	*B. licheniformi*s (T); ATCC 14580; DSM 13	99.3	CP000002
	C	2	*B. licheniformi*s (T); ATCC 14580; DSM 13	98.9	CP000002
	D	1	*B. megaterium* (T); IAM 13418	97.8	D16273
	E	1	*B. megaterium* (T); IAM 13418	98.2	D16273
	F	2	*B. megaterium*; R1ma	99.9	DQ530511
	G	1	*B. circulans* (T); ATCC 4513	99.1	AY724690
	H	1	*B. pumilus* (T); ATCC 7061	98.4	AY876289
	I	2	*B. thuringiensi*s (T); IAM 12077	98.4	D16281
	J	1	*B. altitudinis* (T); type strain:41KF2b	98.8	AJ831842
	K	1	*Sporosarcina koreensis* strain F73	99	NR_043526
	L	1	*B. aquimaris*; VZ‐24	99	HQ386704
	M	1	*B. altitudinis* (T); type strain:41KF2b	100	AJ83184
	N	1	*B. pseudomycoide*s; DSM 12443	100	JX548925
	O	1	*B. simplex* (T); DSM 1321T	99	AJ439078
	P	1	*Solibacillus silvestris* StLB046	99.8	AP012157
Farm 3, Summer	A	1	*B. licheniformi*s (T); ATCC 14580	98.4	CP000002
	B	2	*B. licheniformis* 9945A	99.8	CP005965
	C	2	*B. pumilus* (T); ATCC 7061	99.3	AY876289
	D	4	*B. pumilus* (T); ATCC 7061	99.7	AY876289
	E	1	*B. pumilus*; ML568	99.4	KC692176
	F	1	*B. megaterium*; GSP10	100	AY505510
	G	1	*B. cereus* (T); ATCC 14579	98.6	AE016877
	H	1	*B. cereus* (T); ATCC 14579	97.2	AE016877
	I	1	*B. thuringiensis*; W18	98.6	EF685168
	J	4	*Paenibacillus apiarius*; JF24	98.5	KC172000
	K	1	*B. clausii* KSM‐K16	100	AP006627
	L	1	*Bhargavaea beijingensi*s; KJ‐W4	99.3	JQ799102
	M	3	*B. simplex*; type strain: LMG 11160	100	AJ628743
	N	4	*B. altitudinis* (T); type strain:41KF2b	100	AJ831842
	O	1	*Rummeliibacillus pycnus* (T);NBRC 101231	96.7	AB271739
Farm 4, Winter	A	3	*B. cereus* (T); ATCC 14579	98.5	AE016877
	B	2	*B. megaterium* (T); IAM 13418	98.2	D16273
	C	3	*B. altitudinis* (T); type strain:41KF2b	99.6	AJ831842
Farm 4, Summer	A	1	*B. licheniformis* (T); ATCC 14580;	98	CP000002
	B	1	*B. megaterium* (T); IAM 13418	97.8	D16273
	C	9	*B. pumilus* (T); ATCC 7061	99	AY876289
	D	1	*B. thuringiensis* (T); IAM 12077	99.3	D16281
	E	1	*B. thuringiensis* (T); IAM 12077	99.3	D16281
	F	1	*B. thuringiensis* (T); IAM 12077	99.3	D16281
	G	1	*Paenibacillus telluris* (T); PS38	96.1	HQ257247
	H	1	*Paenibacillus barengoltzii* (T); SAFN‐016	95.7	AY167814
	I	1	*B. stratosphericus* (T) 41KF2a	99.4	AJ831841
	J	1	*B. pseudomycoides*; DSM 12442	98.6	AM747226
	K	2	*B. altitudinis* (T); type strain:41KF2b	99.1	AJ831842
	L	2	*B. altitudinis* (T); type strain:41KF2b	95	AJ831842
	M	6	*B. altitudinis* (T); type strain:41KF2b	99.6	AJ831842

Sequence similarity to various bacterial type strains ispresented.

a The similarity has been confirmed by 16S rRNA sequencing and maximum identity is depicted in percentage.

b Each unique ERIC pattern per farm per sampling event was allocated a separate letter (A to P), depending on the number of ERIC patterns identified from four farms in each sampling event.

**Table 2:** Diversity of anaerobic spore‐forming bacteria isolated from four Waikato farms over two seasons

Farm, Seasons	Eric Type^b^	Number of isolates	Isolate ID^c^	Closest‐related taxonomically described species	Maximum Identity (%)^a^	Genbank Accession Number
Farm 1, Winter	A	14	DE1. 6,8,10,15,16,17,19,22,27,29,38,44,45,46	*C. botulinum* B1 strain Okra	98.9	CP000939
	B	5	DE1. 20.2,33,37,39,42	*C. botulinum* B str. Eklund 17B	99.9	CP001056
	C	2	DE1. 7,18	*C. butyricum* CGS6	99	AY540110
	D	5		*C. cochlearium* JCM 1384	98.8	LC007105
	E	6		*C. cochlearium* DSN5	88	KC331172
	F	5		*C. cadaveris* JCM 1392	99.8	AB542932
	G	15		*C. bifermentans* ATCC 638	97.6	AB075769
Farm 1, Summer	A	21		*C. perfringens* (T); ATCC 13124	99.8	CP000246
	B	2	JDE1.16.1,26	*C. botulinum* B str. Eklund 17B	99	CP001056
	C	1	JDE1.21	*C. sporogenes* (T); ATCC3584	99	X68189
	D	10		*C. senegalence* JC122	99.8	NR_125591
	E	9		*C. glycolicum* (T); DSM 1288	95.4	X76750
Farm 2, Winter	A	1	DE2.33	*C. botulinum* B str. Eklund 17B	99.8	CP001056
	B	1	DE2. 37	*C. butyricum* RCEB	99.7	EU621841
	C	1	DE2. 11	*C. sporogenes* (T); ATCC3584	99	X6818
	D	1	DE2. 29	*C. sporogenes* (T); ATCC3584	100	X6818
	E	1		*C. paraputrificum* ATCC 25780	97.1	X75907
	F	1		*C. senegalence* JC122	99.4	NR_125591
	G	3		*C. glycollicum* (T); DSM 1288	95.8	X76750
	H	3		*C. cadaveris* JCM 1392	100	AB542932
	I	20		*C. bifermentans* ATCC 638	97.7	AB075769
Farm 2, Summer	A	12		*C. perfringens* (T); ATCC 13124	99.4	CP000246
	B	1		*C. perfringens* (T); ATCC 13124	99.2	CP000246
	C	1	JDE2. 14	*C. butyricum* (T); VPI3266	100	AJ458420
	D	1		Swine manure bacterium RT‐8B	97.3	AY167950
	E	1		*C. bifermentans* (T); ATCC 638	95.6	AB075769
Farm 3, Winter	A	1		*C. perfringens* (T); ATCC 13124	99.3	CP000246
	B	5		*C. perfringens* (T); ATCC 13124	99.8	CP000246
	C	1	DE3. 6	*C. butyricum* (T); VPI3266	99.9	AJ458420
	D	2	DE3. 5,7	*C. sporogenes* (T); ATCC3584	99.4	X68189
Farm 3, Summer	A	17		*C. perfringens* (T); ATCC 13124	99.3	CP000246
	B	13		*C. bifermentans* (T); ATCC 638	97.6	AB075769
Farm 4, Winter	A	5	DE4.9,41,22,23,24	*C. botulinum* B1 strain Okra	99	CP000939
	B	5	DE4. 7,18,27,28,40	*C. butyricum* (T); VPI3266	96.7	AJ458420
	C	2	DE4. 32, 27	*C. sporogenes* subsp. *tusciae*	99.4	AM237439
	D	1		*C. tertium* (T); DSM 2485	99.1	Y18174
	E	14		*C. cadaveris* (T); JCM 1392	99.7	AB542932
	F	3		Swine manure bacterium RT‐8B	99	AY167950
Farm 4, Summer	A	1	JDE4. 7	*C. botulinum* B1 strain Okra	98	CP000939
	B	1	JDE4. 10	*C. sporogenes* (T); ATCC3584	93	X68189
	C	1		*C. swellfunianum* strain S11‐3‐10	97.5	NR_126179
	D	8		*Candidatus Clostridium anorexicamassiliense* AP5	98.9	JX101686
	E	6		*C. bifermentans* (T); ATCC 638	97.7	AB075769
	F	3		*C. thiosulfatireducens*; E2_0603	98.3	EF153922

Sequence similarity to various anaerobic bacterial‐type strains is presented.

a The similarity has been confirmed by 16S rRNA sequencing and maximum identity is depicted in percentage.

b Each unique ERIC pattern per farm per sampling event was allocated a separate letter (A to I), depending on the number of ERIC patterns identified from 4 farms in each sampling event.

c Isolate IDs of sequence types belonging to botulinum group I and II are mentioned which have been used for generating phylogenetic relationship and *C. sporogenes* signature PCR.

### 3.3 Occurrence and diversity of aerobic spore‐forming bacteria in FDE

In total 19 different mesophilic *Bacillus* spp., five different *Paenibacillus* spp. and six non *Bacillus* spp. (identified by 16S rRNA gene closest taxonomically described species) were isolated from the four farms (Table 1 and 3). The most commonly isolated *Bacillus* types were those with 16S rRNA sequences that matched *B. licheniformis* (maximum identity 97.6%–100%), *B. altitudinis* (maximum identity 95%–99.6%), *B. pumilus* (maximum identity 98.5%–99.7%), *B. megaterium* (maximum identity 97.4%–100%) and *B. cereus* (maximum identity 97.2%–98.6%). These *Bacillus* sequence types were found on all farms on at least one of the sampling events. *B. cereus* was found on all four farms, Farms 1, 2 and 3 in summer and Farm 4 in winter. *Paenibacillus* sequence‐type isolates (maximum identity 95.7%–99.7%) were found on all farms during summer but only on Farm 1 in winter.

**Table 3:** Prevalence of aerobic spore‐forming bacteria isolated from FDE from each farm over two seasons

	Percentage of various aerobic spore‐forming bacteria							
	Winter				Summer			
Aerobic spore‐forming bacteria	Farm 1	Farm 2	Farm 3	Farm 4	Farm 1	Farm 2	Farm 3	Farm 4
*B. licheniformis*	35%	84%	28.50%		4%	65.70%	10.70%	3.57%
*B. megatarium*			19.04%	25%	4%		3.57%	3.57%
*B. circulans*	15%	8%	4.70%		4%	2.63%		
*B. pumilus*	10%		4.70%		13%		25%	32%
*B. altitudinis*			9.50%	37.50%	30%		14.28%	35.70%
*B. cereus*				37.50%	4%	5.26%	7.14%	
*B. thermoamylovorans*	5%							
*B. mycoides*	5%							
*B. thuringiensis*	5%		9.50%			2.63%	3.57%	10.70%
*B. clausii*	5%	8%					3.57%	
*B. siralis*	5%							
*B. simplex*			4.70%				10.70%	
*B. aquimaris*			4.70%					
*B. pseudomycoides*			4.70%					3.57%
*B. shacletonii*						2.63%		
*B. idriensis*						2.63%		
*B. amyloliquefaciens*						7.90%		
*B subtilis*						5.26%		
*B. stratosphericus*								3.57%
*Paenibacillus spp*	10%				30%	2.63%	14.28%	7.14
Other non‐Bacillus spp.	5%		9.40%		8%	2.63%	7.14%	
Total count (cfu/ml)	20 × 10^3^	25 × 10^5^	21 × 10^5^	8 × 10^3^	23 × 10^3^	38 × 10^4^	28 × 10^3^	28 × 10^3^

Prevalence was based on the percentage of each bacterial species (identified by 16srRNA sequencing) from the total number of colonies isolated from farm dairy effluent samples collected from each farm.

Similar to spore counts as mentioned earlier, Farms 1 and 3 showed no major difference in the diversity of *Bacillus* species isolated. *Bacillus* species isolated from Farms 2 and 4 were found to be more diverse in summer compared with winter sampling, whereas; not much difference was observed with Farm 1 and Farm 3 (Table 1 and 3). On farms 2 and 4 there were only three predominant *Bacillus* species detected in winter compared with Farms 1 and 3, where at least 10 different *Bacillus* species were isolated. Interestingly, *Bacillus* species isolated from Farm 2 in winter were different to Farm 4 and none of those species were common to both the farms (Table 1).

*Bacillus* strains with a 16S rRNA sequence type most closely matching *B. licheniformis* were the most prevalent and also gave rise to the most ERIC types per farm, followed by *B. altitudinis* (Table 3).

### 3.4 Diversity of anaerobic spore‐forming bacteria in FDE

In total, 17 different mesophilic *Clostridium* spp. (identified by 16S rRNA gene closest taxonomically described species), and no non *Clostridium* spp. were isolated from the 4 farms using the methodology described in this study. *Clostridium* species isolated from Farms 1, 2 and 3 were found to be more diverse in winter compared with summer sampling. The most commonly isolated *Clostridium* sequence types were *C. bifermentans* (maximum identity 97.6%–97.7%), *C. perfringens* (maximum identity 99.2%–99.8%), botulinum Group I (maximum identity 98%–100%) and botulinum Group II (maximum identity 98%–99.9%). Botulinum Group I and Group II like sequence types were isolated from FDE obtained from all farms on at least one sampling event. Sequence types closely related to botulinum Group I (type A and B) representatives were identified on Farms 1 and 4. *C. sporogenes* sequence type with maximum identity of 93%–100% was identified on all farms in winter and on Farms 1 and 4 in summer. Botulinum Group II sequence type, related isolates were identified on Farms 1 and 2 during winter but only on Farm 1 during summer. *C. butyricum* sequence types (maximum identity 99%–100%) was found on all farms in winter but only on Farm 2 in summer. No botulinum Group III‐related isolates were identified on any of the farms. Isolates with the closet sequence type similarity to *C. perfringens* were identified on Farms 1, 2 and 3 during summer and only on Farm 3 during winter, whereas, *C. butyricum* sequence types (maximum identity 96.7%–100%) were identified on all four farms in winter and on Farm 2 in summer. (Table 2).

*C. perfringens, C. sporogenes* and *C. cochlearium* (maximum identity 88%–98.8%) were the only species to be represented by two different ERIC types. Most of *Clostridium* spp. isolated was represented by only one ERIC which may suggest a single source of contamination, however, work needs to be done to confirm it. On the whole, *Clostridium* spp. were found to be less diverse on the four farms compared with *Bacillus* spp. but this may be a consequence of culture enrichment undertaken for *Clostridium* spp. isolation.

### 3.5 Phylogenetic analysis of *Clostridium botulinum*‐like species

**Figure 1:**
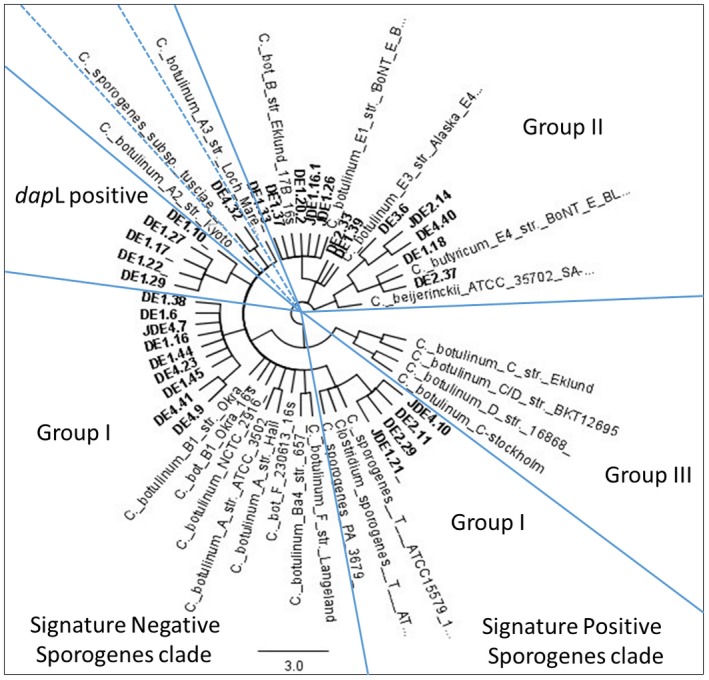
Phylogenetic relationship of farm isolates to botulinum Group I, II and III representatives: A phylogenetic tree was created, using PhyML version 3.1 from the alignment of 16S rRNA gene sequences of representatives of *C. sporogenes* showing the genetic relatedness to botulinum Groups I, II, and III. Specific clades discussed in the text are labeled. DE represents Farm dairy effluent (FDE) sample taken in winter, JDE, FDE sample taken in summer, number represents the farm from which the samples were taken and numbers after decimal represents different isolates

A phylogenetic tree was created, using PhyML tree builder in Geneious version 8.1 from the converted 16S rRNA gene sequence alignments. The tree shows four distinct clustered sets consisting of isolates closely related to botulinum Group I and II, along with few isolates clustering in a completely different set. Four of the isolates clustered in botulinum Group I were found to be closely related to *C. sporogenes* type strain ATCC 15579 where as one isolate was closely related to *C. sporogenes* subsp. *tusciae* which clustered in a different set. The remaining nine isolates clustered under botulinum Group I were found to be closely related to *C. botulinum* B1 strain Okra. Twelve isolates were grouped in botulinum Group II in which five were closely related to *C. butyricum* E4, two were closely related to *C. botulinum* E3 strain Alaska and five isolates were closely related to *C. botulinum* B strain Eklund as well as *C. botulinum* E1 strain BONT E Beluga (Figure 1).

**Figure 2:**
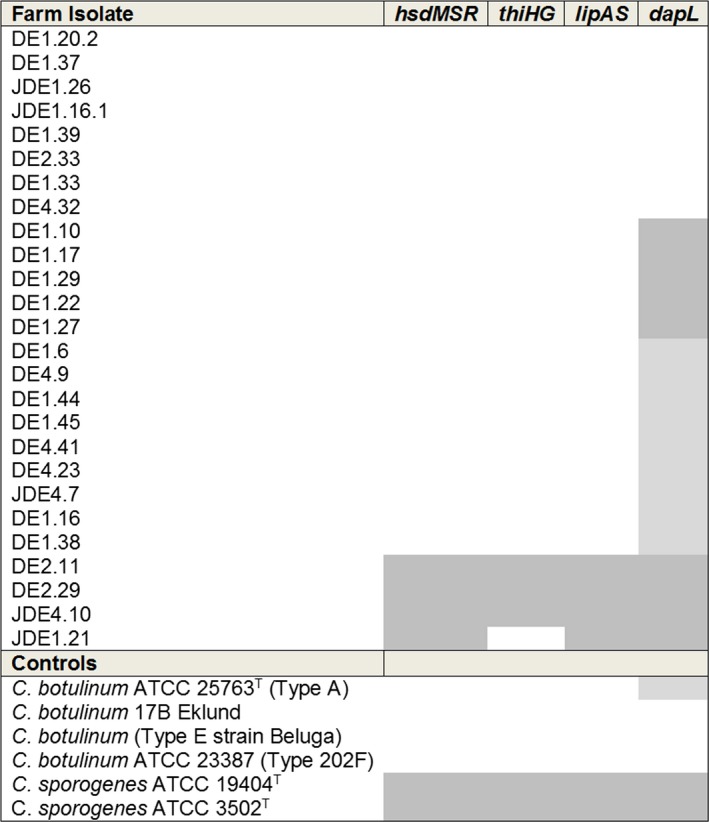
*C. sporogenes* signature PCR: Dark gray highlighted boxes represent isolates PCR positive for the Sporogenes Signature orthologs. Light gray highlighted boxes represent isolates PCR positive for the smaller *dap*L PCR band (500 bp). White boxes were isolates negative for all four Primer sets. Prefix DE, Farm dairy effluent (FDE) sample taken in winter, JDE, FDE sample taken in summer, the number represents the farm from which the samples were taken and numbers after decimal represents different isolates.

Phylogenetic analysis was also performed, using PCR primer sets to four‐specific orthologs from the *C. sporogenes* lineage (Weigand et al., 2015). All farm isolates with 16S rRNA gene sequences closely related to either botulinum Group I and II representatives were tested, using the Sporogenes Signature PCRs (Figure 1 and 2) and the phylogenetic relationship between the Sporogenes Signature Positive isolates compared with Sporogenes Signature Negative, botulinum Group II and Group III isolates are shown in Figure 1.

All four *C. sporogenes* specific orthologs were amplified from farm isolates with the highest 16S rRNA gene sequence identity (93%–100% maximum identity) to *C. sporogenes* PA 3679 and ATCC155791 (Sporogenes Signature Positive Clade) except for sample JD1HE21 (Strain number 21 isolated from Farm1, dairy effluent in January) which was negative for *thiHG*. In addition to the Sporogenes Signature Positive Clade isolates, a *dap*L amplicon was generated, using *C. sporogenes‐*specific primers from isolates DE1HE10, DE1HE17, DE1HE29, DE1HE22, and DE1HE27. However, no amplicons associated with rest of the three *C. sporogenes* specific orthologs were observed from these five strains. The phylogenetic relationship of these isolates was much more closely associated with *C. botulinum* ATCC 3502 type A (98.9% 16S rRNA gene maximum identity). The remaining farm isolates with highest sequence identity to the Group I *C. botulinum* reference strains (Sporogenes Signature Negative Clade) generated a smaller *dap*L amplicon of approximately 500 bp compared to the predicted amplicon of 546 bp. Farm isolates that had sequence similarity closest to the 16S rRNA gene sequence of the botulinum Group II representatives did not generate any amplicons, using the Sporogenes Signature PCR assays (Figures 1 and 2).

Four bacterial isolates (DE2.1, DE2.9, JDE4.10 and JDE1.21) showing sequence similarity to *C. sporogenes* type strain and corresponding to Sporogenes Signature Positive Clade, were found on 3 out of the 4 farms either in summer or winter sampling but not over both seasons. Ten isolates from Farms 1 and 4 corresponded to the Sporogenes Signature Negative Clade, including isolate DE4.32 who's closest sequence type was *C. sporogenes* subsp. *tusciae*. Only one Sporogenes Signature Negative isolate was identified from one farm during the summer sampling event and other isolates were found in winter. All the *dap*L positive isolates were obtained from a single farm (Farm 1) during the winter sampling event (Figure 1).

## 4 Discussion

In New Zealand, FDE is used to irrigate dairy pasture as it is a resource full of nutrients and when managed properly increases pasture production. However, dairy effluent also contains bacteria excreted from the dairy cow and hence provides a good indicator of farm level prevalence of different bacteria. Isolation of environmental spore‐forming bacteria is notoriously challenging. A major challenge has been to separately isolate *Clostridium* spp. and *Bacillus* spp. in anaerobic conditions. This is because *Bacillus* spp. being facultative anaerobes can grow in anaerobic environment. To date, there is no differential agar‐based assay for the detection of SRC species, resulting in a significant percentage of false positives. It is well known that economically significant bacteria such as *C. butyricum*,* C. tyrobutyricum* and *C. sporogenes* are sulphite reducers but there are other species, including *Bacillus* species, such as *B. thuringiensis, B. licheniformis* that will also be enumerated as these species are able to reduce sulphite to sulphide. Also, there is no statistical relationship between SRC counts and the presence of pathogens such as *C. perfringens* and *C. botulinum* in foods (ICMSF 2014). The culture and molecular methods described in this study resulted in improved differential isolation of *Bacillus* and *Clostridium* species. The use of an enrichment step, using prereduced cooked meat glucose broth prior to plating on Shahidi‐ Ferguson Perfringens Agar supplemented with egg yolk, polymyxin B and Kanamycin under anaerobic conditions aided the isolation of only *Clostridium* spp. For example, during the study we did not isolate any facultative anaerobic *Bacillus* strains anaerobically*,* known to be present in the sample. Furthermore, the use of molecular methods such as ERIC genotyping, 16S rRNA sequence analysis and *C. sporogenes* Signature PCR gave further information on the phylogenetic relationship of the *Clostridium* isolates.

Despite regional and methodological differences, the diversity of *Bacillus* species on the four farms was very similar to that seen internationally. In general, *B. licheniformis*,* B. pumilus*, and *B. subtilis* are the predominant mesophilic spore‐forming species (Lukasova, Vyhnalkova, & Pacova, 2001; Sutherland & Murdoch, 1994; Tatzel, Ludwig, Schleifer, & Wallnofer, 1994) and *B. cereus* being the most common psychrotolerant species (Sutherland & Murdoch, 1994). In this study, the most commonly isolated *Bacillus* sequence types were *B. licheniformis* (maximum identity 97.6%–100%), *B. altitudinis* ((maximum identity 95%–99.6%), *B. pumilus* (maximum identity 98.5%–99.7%), *B. megaterium* (maximum identity 97.4%–100%) and *B. cereus* (maximum identity 97.2%–98.6%), with *B. licheniformis* to be the predominant one. These *Bacillus* sequence types were found on all farms on at least one of the sampling events. Our results were found to be concordant to a study which showed a remarkable diversity of aerobic spore‐forming bacteria in dairy farms and *B. licheniformis* amongst the most common ones (Scheldeman et al., 2005). In our study, we isolated a range of aerobic spore‐forming bacteria from farm dairy effluent used for irrigation purposes. McAuley et al., in 2014 also found *Bacillus* group to be the most prevalent bacteria in all the farms tested and highest occurrence was found in soil which was 93% of the samples tested. *Bacillus licheniformis* species have been identified as a prominent food borne pathogens in previous studies. In a study, Rowan et al., 2001 found *B. licheniformis* and *B. megaterium* to produce diarrhoeal enterotoxin in reconstituted infant milk formula. Similarly *B. cereus,* a toxin producing *Bacillus* species have been found in liquid milk and other milk products (Griffiths, 1992). Toxin producing *B. cereus* strains can cause emetic or diarrhoeal food poisoning, while diarrhoeal toxin is produced as a result of spore germination and outgrowth in the small intestine, the emetic toxin is produced by vegetative cells of *B. cereus* growing in the preheat‐treated milk (Kramer & Gilbert, 1989). However, the present cross‐sectional study was carried out investigating the diversity and occurrence of dairy associated spore‐forming bacteria at the farm level and determination of toxicity was not in the scope of this study.

*B. cereus*,* B. licheniformis* and *Paenibacillus* spp. are all psychrotrophic thermophilic sporeformers and a particular problem for the dairy industry. They can grow at refrigeration temperatures and often multiply and sporulate in the bulk milk (Murphy, Lynch, & Kelly, 1999). Their spores can then survive various heat treatments and processing and may go on to cause food poisoning, or reduce the shelf life of pasteurized milk and dairy products (Te Giffel, Beumer, Granum, & Rombouts, 1997). *B. licheniformis* is often the most frequently isolated bacterial contaminant in raw milk (Phillips & Griffiths, 1986; and Crielly, Logan, & Anderton, 1994; Miller et al., 2015). Moreover, some strains of this species have been found to show enhanced growth in skim milk under anaerobic environment (Ronimus et al., 2003).

There is very little data on the occurrence, diversity and seasonal distribution of *Clostridium* species on dairy farms. *Clostridium* species are ubiquitous in soil and presence in feed is common source of contamination of raw milk. Commonly found species are *C. sporogenes*,* C. butryicum*,* C. tyrobutyricum*,* C. disporicum*, and *C. saccharolyticum*. Feligini, Panelli, Sacchi, Ghitti, and Capelli (2014) investigated the occurrence of *Clostridium* spp. in raw milk from Northern Italy. They found that *C. butyricum* and *C. sporogenes* were more abundant in winter, *C. tyrobutyricum* in spring and *C. beijerinckii* in summer. Similarly, in this study, *C. butyricum* and *C. sporogenes* were also found to be abundant in winter than in summer and were representatives from botulinum Groups I and II. From a food safety perspective, *C. perfringens* is considered significant because of the ability of some strains to induce illness (Grass, Gould, & Mahon, 2013) and have also been recurrently identified as the causative agents of mastitis in bovine and other ruminants (Osman, El‐Enbaawy, Ezzeldeen, & Hessein, 2009). *C. perfringens* have been isolated from Australian dairy farms, specifically from milk filters, feces and soil (McAuley et al., in 2014). In this study, *C. perfringens* sequence types were found on all the farms tested on at least one sampling event. Isolates with the closet sequence type similarity to *C. perfringens* were identified on Farms 1, 2, and 3 during summer and only on Farm 3 during winter.

*C. butyricum*, and *C. sporogenes* are known to be associated with spoilage of dairy foods and some strains of *C. butyricum* can produce botulinum like toxin (Le Bourhis et al., 2007; Toyoda, Kobayashi, & Ahiko,^1990^). In this study, *C. butyricum* sequence types (maximum identity 96.7%–100%) were identified on all four farms in winter and on Farm 2 during summer. *C. sporogenes* sequence types (maximum identity 93%–100%) were identified on three farms (2, 3, and 4) during winter but only on Farms 2 and 4 during summer.

Currently there is not enough data to determine if seasonal distribution of *Clostridium* and *Bacillus* species on dairy farms impacts on the risk of contamination of raw milk and downstream processed products. In 2014 MPI (Clostridia (SRC) in New Zealand Bulk Raw Milk) carried out a survey of sulphite reducing clostridia in NZ bulk raw milk which showed that SRC vegetative cells were detected in only one milk sample out of 150 raw milk samples in December and four raw milk samples in February; the spores concentration in all positive test samples being only 1 spore ml^−1^. Consuming low levels of *C. botulinum* spores rarely poses a health issue to individuals older than 1 year old. *C. botulinum* spores are often consumed without ill effect when eating foods such as honey, carrots, potatoes, or smoked fish. Similarly, lower levels of Clostridial spores in dried milk products do not present a health risk. However, the problem arises when the product is rehydrated and kept in anaerobic conditions at nonrefrigeration temperatures, which allow the *C. botulinum* spores to germinate and produce toxins.

The use of plastic‐wrapped and non‐acidified silage as cattle feed has increased the number of botulism outbreaks over the last two decades due to botulinum Groups I‐III in dairy cattle (Böhnel & Gessler, 2013; Lindström, Myllykoski, Sivelä, & Korkeala, 2010). In this study, representatives of botulinum Group I and II were identified on all farms, highlighting the presence of these spores in the dairy farm environment.

Consumption of silage contaminated with spores by cattle was found to be the main reason of raw milk getting contaminated with spore‐forming bacteria (Vissers, Driehuis, Te Giffel, De Jong, & Lankveld, 2006). These spores, survive in the gastrointestinal tract of animals and contaminate the manure by shedding spores in feces which subsequently results in contaminating teats and udder surfaces causing contamination of raw milk during the process of milking (Bergère, Gouet, Hermier, & Mocquot, 1968). Some of the isolates identified in this study may have potential to spoil food or produce toxin. There is always a possibility that if appropriate farming practices are not conducted, these spores may enter raw milk from the dairy environment and can cause problems as described by Driehuis, 2013. This study did not undertake any risk assessment to investigate the possibility of the contamination of raw milk by these isolates. However, this could be a future scope for another valuable study to understand the contamination level of raw milk in dairy farms.

Good farming practices are perhaps the most significant element in controlling spore numbers in raw milk. The use of good quality silage, cleaning, and maintenance of parlor/milking equipment, as well as a stringent udder cleaning and teat preparation before milking are all considered to be good farming practice. Consequently, the management of dairy waste effluent is also a critical component to consider for the reduction in number and spread of spore‐forming bacteria on dairy farms. Furthermore, careful application of dairy waste effluent is particularly important if the farm or neighboring areas are used for wild harvest of foods such as water cress or horticultural operations.

## 5 Future Directions

To investigate seasonal variation in the number of spore‐forming bacteria and their diversity, more samples from different farms will be analyzed during different seasons.

## Conflict of interest

None.

## References

[mbo3457-bib-0001] Ageitos, J. M. , Vallejo, J. A. , Sestelo, A. B. F. , Poza, M. , & Villa, T. G. (2007). Purification and characterization of a milk‐clotting protease from *Bacillus licheniformis* strain USC13. Journal of Applied Microbiology, 103, 2205–2213. 18045403 10.1111/j.1365-2672.2007.03460.x

[mbo3457-bib-0002] Aureli, P. , & Franciosa, G. (2002). Clostridium spp. In: Roginski H et al (eds). Encycl. Dairy Sci. London, UK: Academic Press 456–463.

[mbo3457-bib-0003] Bergère, J. , Gouet, P. , Hermier, J. , & Mocquot, G. (1968). Les Clostridium du groupe butyrique dans les produits laitiers. Annales de Institut Pasteur de Lille, 19, 41–54. 5728589

[mbo3457-bib-0004] Boddinghaus, B. , Wolters, J. , Heikens, W. , & Bottger, E. C. (1990). Phylogenetic analysis and identification of different serovars of *Mycobacterium intracellulare* at the molecular level. FEMS Microbiology Letters, 70, 197–203. 10.1111/j.1574-6968.1990.tb13978.x 2275731

[mbo3457-bib-0005] Böhnel, H. , & Gessler, F. (2013). Presence of *Clostridium botulinum* and botulinum toxin in milk and udder tissue of dairy cows with suspected botulism. The Veterinary Record, 2013(172), 397. 10.1136/vr.100418 23585115

[mbo3457-bib-0006] Burgess, S. A. , Lindsay, D. , & Flint, S. H . (2010). Thermophilic bacilli and their importance in dairy processing. International Journal of Food Microbiology, 144, 215–225. 21047695 10.1016/j.ijfoodmicro.2010.09.027

[mbo3457-bib-0007] Christiansson, A. , Bertilsson, J. , & Svensson, B. (1999). *Bacillus cereus* spores in raw milk: factors affecting the contamination of milk during the grazing period. Journal of Dairy Science, 82, 305–314. 10068952 10.3168/jds.S0022-0302(99)75237-9

[mbo3457-bib-0008] Cocolin, L. , Innocente, N. , Biasutti, M. , & Comi, G. (2004). The late blowing in cheese: a new molecular approach based on PCR and DGGE to study the microbial ecology of the alteration process. International Journal Food Microbiology, 90, 83–91. 10.1016/s0168-1605(03)00296-4 14672833

[mbo3457-bib-0009] Cremonesi, P. , Vanoni, L. , Silvetti, T. , Morandi, S. , & Brasca, M. (2012). Identification of Clostridium beijerinckii, Cl. butyricum, Cl. sporogenes, Cl. tyrobutyricum isolated from silage, raw milk and hard cheese by a multiplex PCR assay. Journal of Dairy Research, 79, 318–323. 22850580 10.1017/S002202991200026X

[mbo3457-bib-0010] Crielly, E. , Logan, N. , & Anderton, A. (1994). Studies on the *Bacillus* flora of milk and milk products. Journal of Applied Bacteriology, 77, 256–263. 7989250 10.1111/j.1365-2672.1994.tb03072.x

[mbo3457-bib-0011] Dodds, K. L . (1993). *Clostridium botulinum* in foods. In: Hauschild A. H. W, Dodds K. L (eds). Clostridium botulinum: ecology and control in foods. Marcel Dekker Inc: New York, pp 53–68.

[mbo3457-bib-0012] Driehuis, F. (2013). Silage and the safety and quality of dairy foods: a review. Agricultural and Food Science, 22, 16–34.

[mbo3457-bib-0013] Dutt, K. , Gupta, P. , Saran, S. , Misra, S. , & Saxena, R. K. (2009). Production of milk‐clotting protease from *Bacillus subtilis* . Applied Biochemistry and Biotechnology, 158, 761–772. 19172237 10.1007/s12010-008-8504-9

[mbo3457-bib-0014] Feligini, M. , Panelli, S. , Sacchi, R. , Ghitti, M. , & Capelli, E . (2014). Tracing the origin of raw milk from farm by using automated ribosomal intergenic spacer analysis (ARISA) fingerprinting of microbiota. Food Control, 50, 51–56.

[mbo3457-bib-0015] Flint, S. , Bremer, P. , & Brooks, J. (1997). Biofilms in dairy manufacturing plant‐description, current concerns and methods of control. Biofouling, 11, 81–97.

[mbo3457-bib-0016] Foschino, R. A. , Galli, A. , & Ottogali, G. (1990). Research on themicroflora of UHT milk. Annual Review of Microbiology, 40, 47–59.

[mbo3457-bib-0017] Fromm, H. I. , & Boor, K. J. (2004). Characterization of pasteurized fluid milk shelf‐life attributes. Journal of Food Science, 69, M207–M214.

[mbo3457-bib-0018] Garde, S. , Gaya, P. , Arias, R. , & Nuñez, M. (2012). Enhanced PFGE protocol to study the genomic diversity of *Clostridium* spp. isolated from Manchego cheeses with late blowing defect. Food Control, 28, 392–399.

[mbo3457-bib-0019] GenBank (http://www.ncbi.nlm.nih.gov/genbank/)

[mbo3457-bib-0020] Gerhardt, P. , & Marquis, R. E. (1989). Spore thermoresistance mechanisms In Smith I., Slepecky R. A., & Setlow P. (Eds.), Regulation of prokaryotic development (pp. 43–63). Washington, D.C: American Society for Microbiology.

[mbo3457-bib-0021] Ghelardi, E. , Celandroni, F. , Salvetti, S. , Barsotti, C. , Baggiani, A. , & Senesi, S . (2002). Identification and characterization of toxigenic *Bacillus cereus* isolates responsible for two food‐poisoning outbreaks. FEMS Microbiology Letters, 208, 129–134. 11934506 10.1111/j.1574-6968.2002.tb11072.x

[mbo3457-bib-0022] Grass, J. E. , Gould, L. H. , & Mahon, B. E. (2013). Epidemiology of foodborne disease outbreaks caused by *Clostridium perfringens*, United States, 1998–2010. Foodborne Pathogens and Disease, 10, 131–136. 23379281 10.1089/fpd.2012.1316 PMC4595929

[mbo3457-bib-0023] Griffiths, M. W. (1992). Bacillus cereus in liquid milk and other milk products. Bulletin of the International Dairy Federation, 275, 36–39.

[mbo3457-bib-0024] Griffiths, M. W. , & Phillips, J. D. (1990). Incidence, source and some properties of psychrotrophic Bacillus found in raw and pasteurised milk. International Journal of Dairy Technology, 43, 62–66.

[mbo3457-bib-0025] Griffiths, M. , Phillips, J. , West, I. , & Muir, D. (1988). The effect of extended low‐temperature storage of raw milk on the quality of pasteurized and UHT milk. Food Microbiology, 5, 75–87.

[mbo3457-bib-0026] Guillaume‐Gentil, O. , Scheldeman, P. , Marugg, J. , Herman, L. , Joosten, H. , & Heyndrickx, M. (2002). Genetic heterogeneity in *Bacillus sporothermodurans* as demonstrated by ribotyping and repetitive extragenic palindromic‐PCR fingerprinting. Applied and Environmental Microbiology, 68, 4216–4224. 12200268 10.1128/AEM.68.9.4216-4224.2002 PMC124129

[mbo3457-bib-0027] Guinebretiere, M. H. , Girardin, H. , Dargaignaratz, C. , Carlin, F. , & Nguyen‐The, C . (2003). Contamination flows of *Bacillus cereus* and spore‐forming aerobic bacteria in a cooked, pasteurised and chilled zucchini purée processing line. International Journal of Food Microbiology, 82, 223–232. 12593925 10.1016/s0168-1605(02)00307-0

[mbo3457-bib-0028] Hanson, M. , Wendorff, W. , & Houck, K. (2005). Effect of heat treatment of milk on activation of *Bacillus* spores. Journal of Food Protection, 68, 1484–1486. 16013392 10.4315/0362-028x-68.7.1484

[mbo3457-bib-0029] Hodcroft, E . (2013). TreeCollapserCL 4. http://emmahodcroft.com/TreeCollapseCL.html3

[mbo3457-bib-0030] Huck, J. R. , Woodcock, N. H. , Ralyea, R. D. , & Boor, K. J. (2007). Molecular subtyping and characterization of psychrotolerant endospore‐forming bacteria in two New York State fluid milk processing systems. Journal of Food Protection, 70, 2354–2364. 17969618 10.4315/0362-028x-70.10.2354

[mbo3457-bib-0031] ICMSF . (2014). Usefulness of Testing for Clostridium botulinum in Powdered Infant Formula and Dairy‐based Ingredients for Infant Formula. ICMSF.

[mbo3457-bib-0032] Julien, M.‐C. , Dion, P. , Lafreniere, C. , Antoun, H. , & Drouin, P. (2008). Sources of clostridia in raw milk on farms. Applied and Environment Microbiology, 74, 6348–6357. 10.1128/AEM.00913-08 PMC2570301 18757576

[mbo3457-bib-0033] Kearse, M. , Moir, R. , Wilson, A. , Stones‐Havas, S. , Cheung, M. , Sturrock, S. , … Drummond, A. (2012). Geneious Basic: an integrated and extendable desktop software platform for the organization and analysis of sequence data. Bioinformatics, 28, 1647–1649. 22543367 10.1093/bioinformatics/bts199 PMC3371832

[mbo3457-bib-0034] Klijn, N. , Herman, L. , Langeveld, L. , Vaerewijck, M. , Wagendorp, A. , Huemer, I. , & Weerkamp, A. (1997). Genotypical and phenotypical characterization of *Bacillus sporothermodurans* strains surviving UHT sterilization. International Dairy Journal, 7, 421–428.

[mbo3457-bib-0035] Klijn, N. , Nieuwendorf, F. F. J. , Hoolwerf, J. D. , van derWaals, C. B. , & Weerkamp, A. H. (1995). Identification of *Clostridium butyricum* as the causative agent of late blowing in cheese by species–species PCR amplification. Applied and Environmental Microbiology, 61, 2919–2924. 7487024 10.1128/aem.61.8.2919-2924.1995 PMC167568

[mbo3457-bib-0036] Kramer, J. M. , & Gilbert, R. J. (1989). *Bacillus cereus* and other *Bacillus* species. Foodborne Bacterial Pathogens, 19, 21–70.

[mbo3457-bib-0037] Le Bourhis, A. G. , Doré, J. , Carlier, J. P. , Chamba, J. F. , Popoff, M. R. , & Tholozan, J. L. (2007). Contribution of C. beijerinckii and C. sporogenes in association with C. tyrobutyricum to the butyric fermentation in Emmental type cheese. International Journal of Food Microbiology, 113, 154–163. 17169455 10.1016/j.ijfoodmicro.2006.06.027

[mbo3457-bib-0038] Lindström, M. , Myllykoski, J. , Sivelä, S. , & Korkeala, H. (2010). Clostridium botulinum in cattle and dairy products. Critical Reviews in Food Science and Nutrition, 50, 281–304. 20301016 10.1080/10408390802544405

[mbo3457-bib-0039] Lukasova, J. , Vyhnalkova, J. , & Pacova, Z. (2001). *Bacillus* species in raw milk and in the farm environment. Milchwissenschaft, 56, 609–611.

[mbo3457-bib-0040] McAuley, C. M. , McMillan, K. , Moore, S. C. , Fegan, N. , & Fox, E. M. (2014). Prevalence and characterization of foodborne pathogens from Australian dairy farm environments. Journal of Dairy Science, 97, 7402–7412. 25282417 10.3168/jds.2014-8735

[mbo3457-bib-0041] Miller, R. A. , Kent, D. J. , Watterson, M. J. , Boor, K. J. , Martin, N. H. , & Wiedmann, M. (2015). Spore populations among bulk tank raw milk and dairy powders are significantly different. Journal of Dairy Science, 98, 8492–8504. 26476952 10.3168/jds.2015-9943

[mbo3457-bib-0042] Murphy, P. M. , Lynch, D. , & Kelly, P. M. (1999). Growth of thermophilic spore forming bacilli in milk during the manufacture of low heat powders. International Journal of Dairy Technology, 52, 45–50.

[mbo3457-bib-0043] New Zealand Ministry of Primary Industries (MPI) . (2014). Clostridia (SRC) in New Zealand Bulk Raw Milk MPI Technical Paper No: 2014/40 Prepared by Food Risk Assessment group March.

[mbo3457-bib-0044] Osman, K. M. , El‐Enbaawy, M. I. , Ezzeldeen, N. A. , & Hessein, H. M. G. (2009). Mastitis in dairy buffalo and cattle in Egypt due to Clostridium perfringens: prevalence, incidence, risk factors and costs”. Revue Scientifique et Technique (International Office of Epizootics), 28, 975–986. 20462154 10.20506/rst.28.3.1936

[mbo3457-bib-0045] PATRIC Pathosystems Resource Integration Centre https://www.patricbrc.org.

[mbo3457-bib-0046] Peck, M. W. (2006). *Clostridium botulinum* and the safety of minimally heated, chilled foods: an emerging issue? Journal of Applied Microbiology, 101, 556–570. 16907806 10.1111/j.1365-2672.2006.02987.x

[mbo3457-bib-0047] Phillips, J. D. , & Griffiths, M. W. (1986). Factors contributing to the seasonal variation of *Bacillus* spp. in pasteurized dairy products. Journal of Applied Bacteriology, 61, 275–285. 3781939 10.1111/j.1365-2672.1986.tb04288.x

[mbo3457-bib-0048] Prevot, A . (1948). Recherches sur la réduction des sulfates et des sulfites minéraux par les bactéries anaérobies. Paris, France: Annales de L Institut PasteurMasson Editeur.

[mbo3457-bib-0049] Rammer, C. (1996). Quality of grass silage infected with spores of *Clostridium tyrobutyricum* . Grass & Forage Science, 51, 88–95.

[mbo3457-bib-0050] Ranieri, M. L. , Huck, J. R. , Sonnen, M. , Barbano, D. M. , & Boor, K. J. (2009). High temperature, short time pasteurization temperatures inversely affect bacterial numbers during refrigerated storage of pasteurized fluid milk. Journal of Dairy Science, 92, 4823–4832. 19762797 10.3168/jds.2009-2144

[mbo3457-bib-0051] Ranieri, M. L. , Ivy, R. A. , Mitchell, W. R. , Call, E. , Masiello, S. N. , Wiedmann, M. , & Boor, K. J. (2012). Real‐time PCR detection of *Paenibacillus* spp. in raw milk to predict shelf life performance of pasteurized fluid milk products. Applied and Environment Microbiology, 78, 5855–5863. 10.1128/AEM.01361-12 PMC3406147 22685148

[mbo3457-bib-0052] Ronimus, R. S. , Parker, L. E. , Turner, N. , Poudel, S. , Rückert, A. , & Morgan, H. W. (2003). A RAPD based comparison of thermophilic bacilli from milk powders. International Journal of Food Microbiology, 85, 45–61. 12810270 10.1016/s0168-1605(02)00480-4

[mbo3457-bib-0053] Rowan, N. J. , Deans, K. , Anderson, J. G. , Gemmell, C. G. , Hunter, I. S. , & Chaithong, T. (2001). Putative virulence factor expression by clinical and food isolates of *Bacillus* spp. after growth in reconstituted infant milk formulae. Applied and Environment Microbiology, 67, 3873–3881. 10.1128/AEM.67.9.3873-3881.2001 PMC93104 11525980

[mbo3457-bib-0054] Scheldeman, P. , Pil, A. , Herman, L. , De Vos, P. , & Heyndrickx, M. (2005). Incidence and diversity of potentially highly heat‐resistant spores isolated at dairy farms. Applied and Environmental Microbiology, 71, 1480–1494. 15746351 10.1128/AEM.71.3.1480-1494.2005 PMC1065131

[mbo3457-bib-0055] Shahidi, S. A. , & Ferguson, A. R. (1991). New quantitative, qualitative, and confirmatory media for rapid analysis of food for *Clostridium perfringens* . Applied Microbiology, 21, 500–506. 10.1128/am.21.3.500-506.1971 PMC377211 4324195

[mbo3457-bib-0056] Slaghuis, B. A. , Te Giffel, M. C. , Beumer, R. R. , & André, G. (1997). Effect of pasturing on the incidence of *Bacillus cereus* spores in raw milk. International Dairy Journal, 7, 201–205.

[mbo3457-bib-0057] Sutherland, A. D. , & Murdoch, R. (1994). Seasonal occurrence of psychrotrophic *Bacillus* species in raw milk, and studies on the interactions with mesophilic *Bacillus* sp. International Journal of Food Microbiology, 21, 279–292. 8043347 10.1016/0168-1605(94)90058-2

[mbo3457-bib-0058] Tatzel, R. , Ludwig, W. , Schleifer, K. H. , & Wallnofer, P. R. (1994). Identification of *Bacillus* strains isolated from milk and cream with classical and nucleic acid hybridization methods. Journal of Dairy Research, 61, 529–535. 7829756 10.1017/s0022029900028454

[mbo3457-bib-0059] Te Giffel, M. , Beumer, R. , Granum, P. , & Rombouts, F. (1997). Isolation and characterisation of *Bacillus cereus* from pasteurised milk in household refrigerators in the Netherlands. International Journal of Food Microbiology, 34, 307–318. 9039575 10.1016/s0168-1605(96)01204-4

[mbo3457-bib-0060] Toyoda, S. , Kobayashi, Y. , & Ahiko, K. (1990). Isolation and characterization of clostridia from Gouda cheese with late gas‐blowing. Japanese Journal of Zootechnical Science, 61, 591–598.

[mbo3457-bib-0061] Turnbull, P. C. , Jackson, P. J. , Hill, K. K. , Keim, P. , Kolstø, A. B. , & Beecher, D. J . (2002). Long standing taxonomic enigmas within the ‘*Bacillus cereus* group’ are on the verge of being resolved by far‐ reaching molecular developments: forecasts on the possible outcome by an adhoc team In Berkeley R., Heyndrickx M., Logan N. & DeVos P. (Eds.), Applications and Systematics of Bacillus and Relatives (pp. 23–36). Oxford: Blackwell Publishing.

[mbo3457-bib-0062] Versalovic, J. , Koueth, T. , & Lupski, J. R. (1991). Distribution of repetitive DNA sequences in eubacteria and application to fingerprinting of bacterial genomes. Nucleic Acid Research, 19, 6823–6831. 10.1093/nar/19.24.6823 PMC329316 1762913

[mbo3457-bib-0063] Vissers, M. M. M. , Driehuis, F. , Te Giffel, M. C. , De Jong, P. , & Lankveld, J. M. G. (2006). Improving farm management by modeling the contamination of farm tank milk with butyric acid bacteria. Journal of Dairy Science, 89, 850–858. 16507677 10.3168/jds.S0022-0302(06)72148-8

[mbo3457-bib-0064] Vissers, M. , Driehuis, F. , Te Giffel, M. , De Jong, P. , & Lankveld, J. (2007a). Concentrations of butyric acid bacteria spores in silage and relationships with aerobic deterioration. Journal of Dairy Science, 90, 928–936. 17235169 10.3168/jds.S0022-0302(07)71576-X

[mbo3457-bib-0065] Vissers, M. , Driehuis, F. , Te Giffel, M. , De Jong, P. , & Lankveld, J. (2007b). Minimizing the level of butyric acid bacteria spores in farm tank milk. Journal of Dairy Science, 90, 3278–3285. 17582112 10.3168/jds.2006-798

[mbo3457-bib-0066] Vissers, M. , Te Giffel, M. , Driehuis, F. , De Jong, P. , & Lankveld, J. (2007). Minimizing the level of *Bacillus cereus* spores in farm tank milk. Journal of Dairy Science, 90, 3286–3293. 17582113 10.3168/jds.2006-873

[mbo3457-bib-0067] Weenk, G. , Van den Brink, J. , Struijk, C. , & Mossel, D. (1995). Modified methods for the enumeration of spores of mesophilic *Clostridium* species in dried foods. International Journal of Food Microbiology, 27, 185–200. 8579989 10.1016/0168-1605(94)00164-2

[mbo3457-bib-0068] Weigand, M. R. , Pena‐Gonzalez, A. , Shirey, T. B. , Broeker, R. G. , Ishaq, M. K. , Konstantinidis, K. T. , & Raphael, B. H . (2015). Implications of Genome‐Based Discrimination between *Clostridium botulinum* Group I *and Clostridium sporogenes* Strains for Bacterial Taxonomy. Applied and Environment Microbiology, 81, 5420–5429. 10.1128/AEM.01159-15 PMC4510194 26048939

[mbo3457-bib-0069] Weijtens, M. J. B. M. , Reinders, R. D. , Urlings, H. A. P. , & Van der Plas, J. (1999). *Campylobacter* infections in fattening pigs; excretion pattern and genetic diversity. Journal of Applied Microbiology, 86, 63–70. 10030012 10.1046/j.1365-2672.1999.00636.x

